# The impact of *Paulownia*–buckwheat intercropping on the biodiversity of different living biota

**DOI:** 10.1038/s41598-026-58572-1

**Published:** 2026-06-20

**Authors:** Marek Liszewski, Anna Jama-Rodzeńska, Małgorzata Woźniak, Elżbieta Gębarowska, Sylwia Siebielec, Jacek Twardowski, Iwona Gruss, Ewa Tendziagolska, Piotr Kuc, Paweł Chorbiński, Dariusz Zalewski, Bernard Gałka

**Affiliations:** 1 Institute of Agroecology and Plant Production, Wroclaw University of Environmental and Life Sciences, Grunwaldzki Square 24a, 50-363 Wroclaw, Poland; 2 Department of Microbiology, Institute of Soil Science and Plant Cultivation–State Research Institute, Czartoryskich 8, 24-100 Pulawy, Poland; 3 Department of Plant Protection, Wroclaw University of Environmental and Life Sciences, Grunwaldzki Square 24a, 53-363 Wroclaw, Poland; 4 Department of Epizootiology with Exotic Animal and Bird Clinic, Wroclaw University of Environmental and Life Sciences, Grunwaldzki Square 45, 50-366 Wrocław, Poland; 5 Department of Genetics, Plant Breeding and Seed Production, Wrocław University of Environmental and Life Sciences, Grunwaldzki Square 24a, 50-363 Wroclaw, Poland; 6 Institute of Soil Science, Plant Nutrition and Environmental Protection, Wroclaw University of Environmental and Life Sciences, Grunwaldzki Square 53, 50-357 Wroclaw, Poland

**Keywords:** Beekeeping value, Buckwheat, *Paulownia*, Mesofauna, Soil microorganisms, Weeds, Ecology, Ecology, Microbiology, Plant sciences

## Abstract

Intercropping has emerged as a promising strategy to improve agroecosystem biodiversity and mitigate some adverse effects associated with intensive monoculture systems. This study evaluated weed infestation and biodiversity responses in a *Paulownia*–buckwheat intercropping system compared with buckwheat monoculture under the environmental conditions of southwestern Poland. Weed species composition, abundance, and biomass were assessed at different growth stages of buckwheat. Selected agroecosystem components, including soil microorganisms, soil mesofauna, and pollinator abundance, were evaluated. The intercropping system increased Collembola diversity and Acari abundance, while higher diversity and richness of segetal plant species were also observed compared with monoculture. Higher bacterial abundance and dehydrogenase activity were recorded under intercropping, whereas fungal community composition remained generally stable between cultivation systems. Several melliferous weed species were identified within the intercropping system, potentially supporting pollinator activity. Although no statistically significant differences were observed in pollinator-related parameters, nectar productivity and sugar availability tended to be higher under intercropping conditions, suggesting that the *Paulownia*–buckwheat system may contribute to maintaining pollinator activity and supporting agroecosystem biodiversity. No significant differences in buckwheat yield or biometric traits were found between cultivation systems. Overall, the results indicate that *Paulownia*–buckwheat intercropping may enhance selected components of agroecosystem biodiversity without reducing crop productivity.

**Supplementary Information:**

The online version contains supplementary material available at https://doi.org/10.1038/s41598-026-58572-1.

## Introduction

Intercropping is considered a sustainable agricultural practice that may enhance biodiversity and support ecosystem services while reducing the negative effects of intensive monoculture systems ^1,2^. These benefits are often attributed to ecological mechanisms such as complementary resource use, modified competition for light, water and nutrients, and improved nutrient cycling within agroecosystems ^1,2^. In agroecosystems, weeds are not only competitors of crop plants but may also contribute to trophic interactions, pollinator support, and habitat heterogeneity ^3–5^. Agricultural management practices strongly influence weed communities and associated soil and aboveground organisms, including soil mesofauna and microorganisms ^6^. Changes in weed communities may therefore affect aboveground–belowground interactions by modifying pollinator resources, litter inputs, and soil biological activity ^5,6^. In agroforestry systems, diversified vegetation structure may additionally modify weed assemblages and create more heterogeneous habitats for soil organisms. Tree components can also alter microclimatic conditions through partial shading, reduced soil evaporation, and changes in soil moisture, thereby influencing the diversity and functioning of plant and soil communities ^1,6^.

From an ecological perspective, maintaining diverse weed communities may support agroecosystem functioning and biodiversity conservation^3,4,7^. Beyond their direct contribution to plant diversity, weed communities may provide food resources and habitat for pollinators, support higher trophic levels, and contribute organic residues that stimulate soil microbial activity and nutrient cycling ^3,5,6^. However, balancing biodiversity enhancement with crop productivity remains a major challenge in modern agriculture. Intercropping systems have been proposed as an approach that may improve ecosystem stability, reduce weed competitiveness, and promote beneficial organisms ^2,8^. By increasing plant diversity and habitat heterogeneity, intercropping may strengthen ecological linkages between aboveground communities, including weeds and pollinators, and belowground communities such as microorganisms and soil mesofauna ^2,8^. In addition to their effects on crop productivity, weeds may also provide ecological functions by supporting pollinators and soil microorganisms ^9,10^. Consequently, evaluating weed communities together with other biological components of agroecosystems may provide a more comprehensive understanding of biodiversity responses to intercropping practices.

Buckwheat is considered a valuable melliferous crop, whereas *Paulownia*-based agroforestry systems are increasingly recognized for their ecological and production potential ^1,11^. *Paulownia* is characterized by rapid growth, high biomass production, extensive root development, and the ability to modify microclimatic conditions through partial shading, which may influence soil moisture, nutrient availability, and the activity of associated organisms ^1,11^. Previous studies demonstrated that intercropping systems may improve soil biological activity, increase pollinator abundance, and support biodiversity in agricultural landscapes ^2,12^. These effects are often associated with enhanced habitat heterogeneity and ecological interactions linking aboveground and belowground components of agroecosystems, including plants, pollinators, soil microorganisms, and mesofauna ^1,12^. However, little is known about the effects of *Paulownia*–buckwheat intercropping on weed communities and soil mesofauna under Central European conditions. This knowledge gap is particularly important because agroforestry systems established in Central Europe are exposed to climatic constraints, including spring frosts, seasonal droughts, and shorter growing seasons, which may influence species interactions and biodiversity responses differently than in regions where *Paulownia* is traditionally cultivated. Previous studies on this experimental system conducted during 2021–2022 growing seasons focused mainly on soil microbiology, pollinator activity, and crop productivity ^11,13^, whereas the biodiversity of segetal flora and soil arthropods under the fields conditions remains insufficiently investigated. Moreover, integrated assessments simultaneously linking weed communities, soil mesofauna, microorganisms, and pollinator-supporting vegetation within *Paulownia*-based intercropping systems remain scarce.

Therefore, the aim of this study was to evaluate weed infestation and biodiversity responses in a *Paulownia*–buckwheat intercropping system compared with buckwheat monoculture. The study was designed to assess both aboveground and belowground components of agroecosystem biodiversity and to improve understanding of the ecological interactions occurring within this agroforestry system. Particular attention was given to weed community structure, soil mesofauna diversity, selected microbiological parameters, pollinator abundance, and crop productivity. By integrating these biological indicators, the study aimed to provide a more comprehensive assessment of biodiversity responses to intercropping under Central European environmental conditions. We hypothesized that intercropping would increase the biodiversity of selected agroecosystem components while maintaining stable buckwheat yield.

## Materials and methods

### Study sites and experimental design

**Fig. 1:**
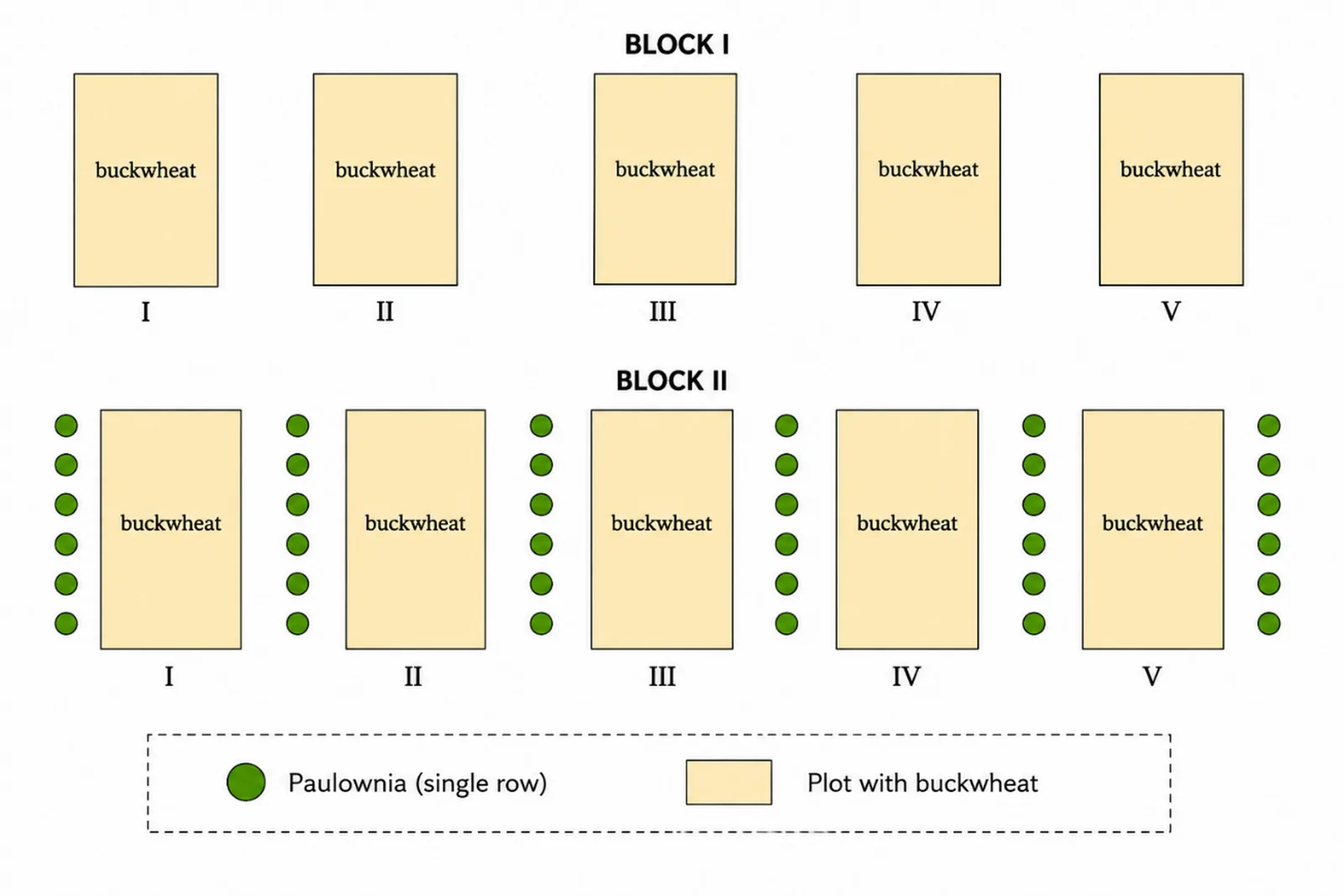
Experimental design for the cultivation of *Paulownia* and buckwheat.

The study site with the experimental factors and general methodology applied in the present study have been described previously by Woźniak et al. (2025) ^13^ and Chorbiński et al. (2024) ^11^ (Fig. 1). Crop management and agrotechnical practices were conducted according to the procedures previously described by Woźniak et al. (2025) ^13^.

### Soil conditions and chemical analysis

Detailed characteristics of the experimental site and soil properties were previously described by Woźniak et al. (2025) ^13^ and Chorbiński et al. (2024) ^11^. Selected physicochemical soil properties determined in 2024 are presented in Table 1. The soil was characterized by slightly acidic to neutral pH and moderate to high nutrient availability. According to the WRB and the Polish Soil Classification systems, the soil was classified as a humus-rich soil.

**Table 1:** Selected physicochemical soil properties at two soil depths in 2024.

Parameter	Spring				Summer			
	0–30		30–60		0–30		30–60	
	AK	AP	AK	AP	AK	AP	AK	AP
pH in KCl (soil pH)	6.1	6.4	6.2	6.7	5.8	5.8	6.0	5.8
Phosphorus P_2_O_5_ (mg/100 g of soil)	19.3	2.4	18.8	12.3	27.1	30.6	26.0	30.9
Potassium K_2_O (mg/100 g of soil)	9.4	23.4	12.6	9.4	19.3	27.1	17.5	24.1
Magnesium MgO (mg/100 g of soil)	16.6	15.7	18.2	18.6	11.4	12.7	11.3	12.8
N min. (g/kg)	0.2	0.2	0.2	0.6	0.6	0.1	0.8	0.8
% C	0.9	0.3	0.4	0.3	0.7	0.3	0.8	1.1

*AK* control (buckwheat monoculture), *AP* intercropping (*Paulownia*–buckwheat intercropping system).

### Weather conditions

Meteorological conditions during the 2024 growing season at the Research and Teaching Station of the Wrocław University of Environmental and Life Sciences were characterized based on temperature, rainfall, and the Selyaninov hydrothermal coefficient (HTC), as previously described by Chorbiński et al. (2024) ^11^. Weather conditions in 2024 differed from the long-term mean (1990–2020), particularly with lower precipitation in April and June and excessive rainfall in August (Table 2). These conditions may have influenced buckwheat growth, development, and harvesting.

**Table 2:** Meteorological conditions and Selyaninov hydrothermal coefficient (HTC) in 2024.

Month	Temperature (°C)		Rainfall (mm)		Selyaninov’s coefficient (K)
	2024	Mean 1990–2020	2024	Mean 1990–2020	2024
Apr	11.3	9.6	13.3	32.8	0.39
May	16.6	14.3	27.6	58.9	0.54
Jun	19.1	17.8	33.8	74.6	0.59
Jul	21.3	19.7	60.0	86.6	0.91
Aug	20.5	19.2	127.7	63.6	2.00
Sep	16.1	14.2	91.7	50.6	1.90
Mean/total (IV–IX)	17.5	15.8	354.1	367.1	–

### Soil moisture measurment

Volumetric soil water content (% vol.) in the upper 55 mm soil layer was measured using an SM150 ThetaProbe moisture sensor connected to a Delta-T HH2 handheld data logger (Delta-T Devices Ltd., Cambridge, UK). Measurements were taken on 15 April, 4 June, 23 July, and 15 October 2024. Soil moisture was determined in the middle of each plot with 20 replicates per treatment.

### Weed community assessment

Weed infestation was evaluated at three growth stages of buckwheat: after emergence (BBCH 12), before harvest (BBCH 85), and four weeks after harvest. Weed species composition and abundance were determined using 0.2 m^2^ sampling plots (five replicates per plot) at BBCH 12 and 0.5 m^2^ sampling plots (two replicates per plot) at later assessment dates. Weed dry biomass was determined before and after harvest.

### Soil mesofauna assessment

Soil was sampled using a circular sampler with a diameter of 10 cm from a depth of 10 cm. Six samples were collected from each plot, resulting in a total of 66 samples collected on each of three sampling dates (June, July, and October). Soil samples were heated in a MacFadyen extractor for seven days, with the temperature gradually increasing from 20 °C to 40 °C. The extracted soil invertebrates extracted from the samples were preserved in 75% ethyl alcohol. The total abundance of Acari and Collembola were assessed through microscope observations. Additionally, Collembola were identified to species or morphotypes, which was used to determine species diversity. The identification key proposed of Hopkin (2007) ^14^ was used.

### Pollinator observations and beekeeping value

Nectar secretion and pollinator abundance in buckwheat were evaluated in June 2024 using the methodology previously described by Chorbiński et al. (2024)^11^. Nectar secretion was determined using the pipette method according to Jabłoński (2002)^15^.

### Soil microbiological analyses

Soil microbiological analyses were performed according to the procedures previously described by Woźniak et al. (2025) ^13^. Soil samples were collected before buckwheat sowing (T1, 15 April 2024), during flowering (T1a), and after harvest (T2, 23 July 2024). Microbial community structure was assessed based on DNA extraction followed by high-throughput sequencing analyses, microbial analyses, fungal identification, and selected soil biological activity parameters, including dehydrogenase activity (DHA) and total glomalin-related soil proteins (T-GRSP). Fungal colonization of buckwheat roots was additionally assessed at flowering (T1a) by isolation and morphological identification of fungi, and the extent of colonization was expressed as the percentage of infected roots.

Soil mesofauna and microbiological analyses were conducted at different sampling times due to differences in the seasonal dynamics and activity patterns of these biological groups.

### Yield and biometric traits

Biometric analyses of buckwheat plants performed in this study followed the methodology previously described by Woźniak et al. (2025) ^13^ and Chorbiński et al. (2024) ^11^.

### Statistical analysis

 The effect of the cultivation system during the study period on biometric traits and buckwheat yield, soil moisture, as well as weed abundance and dry biomass, was evaluated using Student’s t-test in Statistica 13.1 software ^16^. Differences between means were considered significant at the α = 0.05 level. For soil fauna, a generalized linear mixed model (GLMM) was employed to evaluate the effects of treatment (Intercropping vs. Control) and date (June, July, and September), with blocks included as a random effect. The primary groups of mesofauna analyzed included Collembola abundance, Collembola species richness (assessed using the Margalef index), and Acari abundance. The Margalef index was calculated using the formula: D = (S − 1)/(ln(N), where *S* is the total number of species and *N* is the total number of individuals. The appropriate distribution for each analysis was selected based on the Akaike Information Criterion (AIC). Additionally, the Tukey post-hoc test was conducted to visualize the differences between treatments. Mesofauna analyses were performed using SAS University Edition software.

## Results

### Buckwheat and *Paulownia* development phases

High temperatures in the initial period of development (April–June) accelerated the development of buckwheat (Table S1, Supplementary Materials). However, high temperatures and drought during the flowering stage negatively affected flower development and nectar production, which likely reduced pollinator activity. Shortening of the flowering period, it struggled with heat and low humidity, which resulted in a deterioration of nectar production. This resulted in worse bee flights. Shortening of the flowering period and limited plant growth caused by water deficit may have contributed to the low buckwheat yield observed in 2024. Excessive rainfall in August additionally delayed buckwheat ripening and harvest.

In the case of *Paulownia*, temperature conditions were generally favourable for this thermophilic species. However, frost events occurring on 23–24 April damaged flowers and leaves, delayed vegetation development, and disrupted early growth. The extended autumn period subsequently prolonged the vegetation period, resulting long growth duration for *Paulownia* under Polish environmental conditions.

### Soil moisture during growing season

**Fig. 2:**
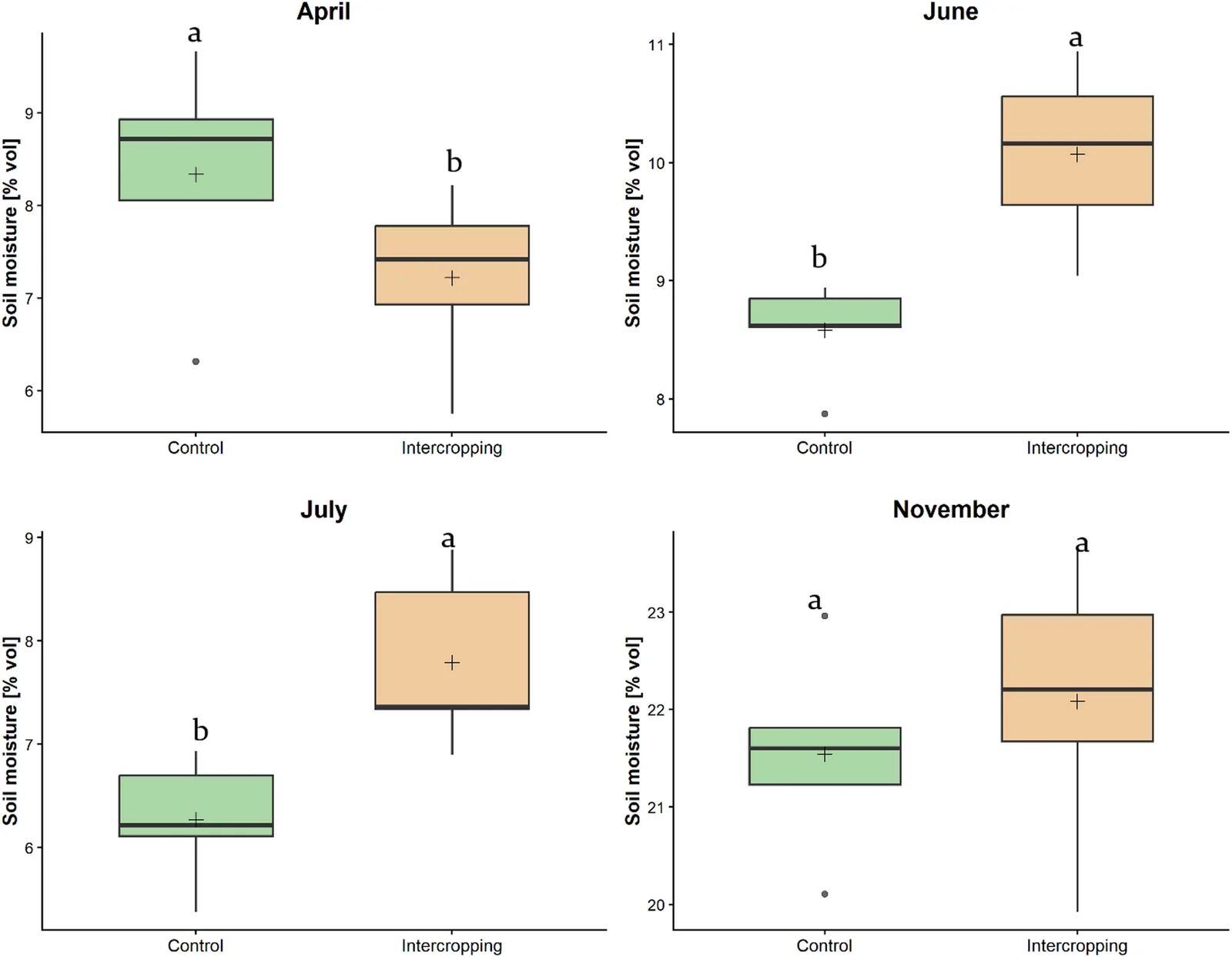
Effect of intercropping on soil moisture at different growth stages of buckwheat and *Paulownia.*

Soil moisture varied between cultivation systems depending on the measurement date and plant developmental stage (Fig. 2). During the first measurement conducted before buckwheat sowing and during *Paulownia* flowering (April), significantly higher soil moisture was recorded in the control plots, with a difference of 1.1 percentage points between treatments. During *Paulownia* crown growth and buckwheat full bloom (June), the surface soil layer in the intercropping plots had noticeably higher moisture content than the control plots, with a difference of 1.4 percentage points. Similar relationships were observed during the third measurement conducted at the buckwheat seed maturity stage (July). Soil moisture in the intercropping treatment reached 7.8% vol., compared with 6.3% vol. in the control treatment. After buckwheat harvest and before the end of the *Paulownia* growing season (October), no significant differences in soil moisture were observed between the cultivation systems.

### Effect of intercropping on mesofauna abundance

**Fig. 3:**
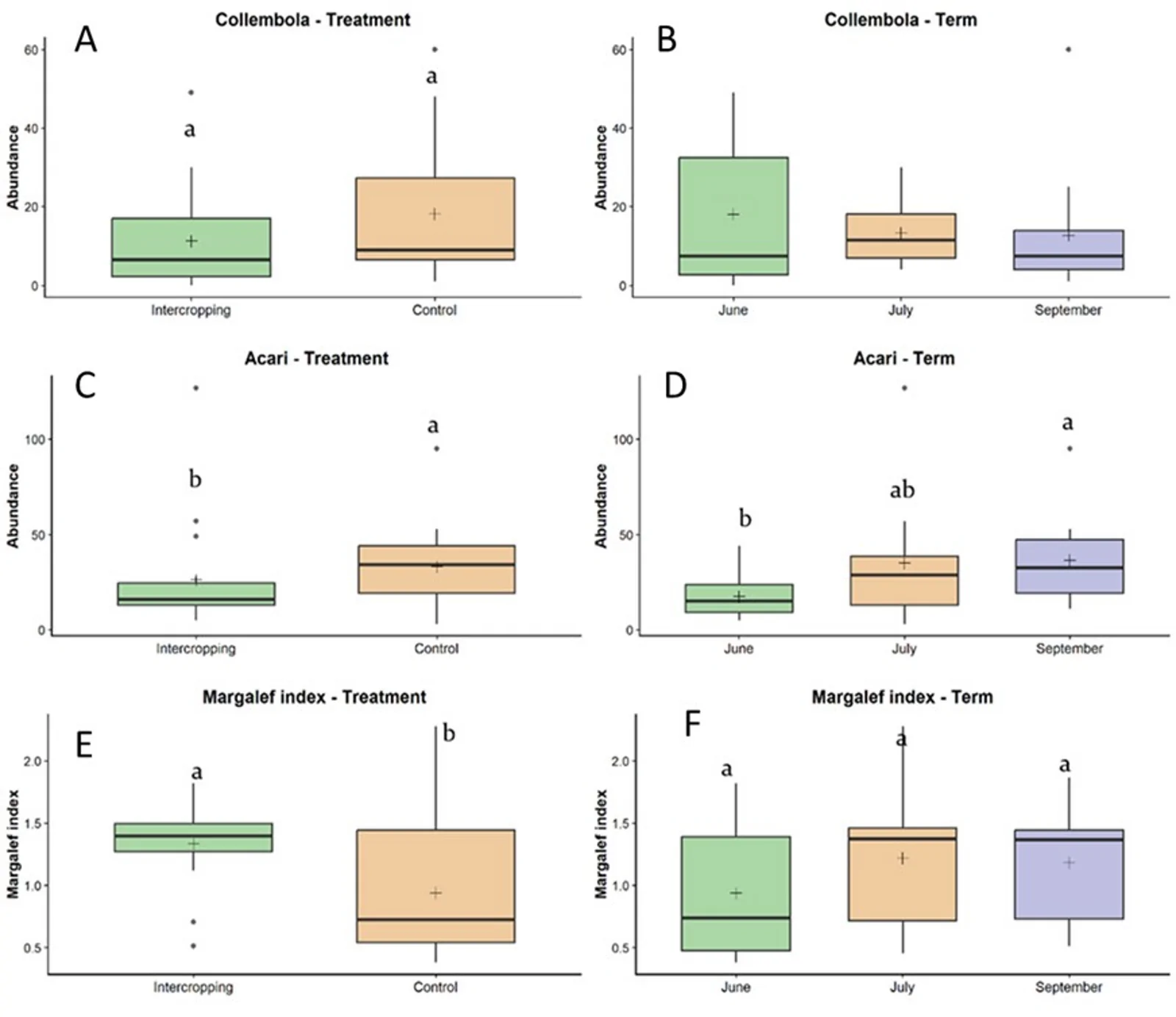
Effect of treatment (intercropping vs. control) and sampling term (June, July, and September) on Collembola abundance (**A**, **B**), Acari abundance (**C**, **D**), and Collembola diversity expressed by the Margalef index (**E**, **F**). Different lowercase letters indicate significant differences between treatments.

No significant differences in Collembola abundance were observed between cultivation systems or sampling dates (Fig. 3A, B). Although slightly higher values were recorded in the control treatment and in July, these differences were not statistically significant.

In contrast, intercropping significantly increased Collembola diversity, as indicated by higher Margalef index values compared with the control treatment (Fig. 3E). No significant differences in the Margalef index were observed among sampling dates (Fig. 3F).

Acari abundance differed significantly between cultivation systems and sampling dates. Higher Acari abundance was recorded in the control treatment than in the intercropping system (Fig. 3C). In addition, Acari abundance increased during the growing season, with the highest values observed in September (Fig. 3D).

### Impact of different cultivation systems on weed infestation in buckwheat

A higher number of weed species was observed in the intercropping system after buckwheat harvest, although differences in total weed abundance and biomass between cultivation systems were generally not statistically significant (Table 3). Despite the slightly higher weed abundance recorded under intercropping conditions, weed dry biomass was lower than in the control treatment, suggesting reduced weed competitiveness in the presence of *Paulownia*.

**Table 3:** Number (no./m^2^) and dry mass of weeds (g) during 3 stages of buckwheat vegetation in 2024.

Specification	Number of weeds at buckwheat emergence (BBCH 12)	Number of weeds at buckwheat BBCH 85	Number of weeds after buckwheat harvest	Dry mass of weeds at buckwheat BBCH 85	Dry mass of weeds after buckwheat harvest
AP	47.7	74.6	72.8	90.2	24.3
AK	57.0	72.4	36.0	171.2	33.5
Test t-Student’s	ns—non-significant *p* = 0.37	ns—non-significant * p* = 0.84	ns—non-significant * p* = 0.08	ns—non-significant * p* = 0.17	ns—non-significant * p* = 0.39

*AK* control (buckwheat monoculture), *AP* intercropping (*Paulownia*–buckwheat intercropping system).

The dominant species of weed were observed: *Chenopodium album, Echinochloa crusgalli*, and *Agropyron repens.* There were no statistically significant differences between the cultivation system and the number of weeds; however, a lower number of weed species can be generally found in the intercropping system (Table S2—S4). Although no statistically significant differences were observed in the abundance or dry biomass of dominant weed species between cultivation systems, lower values were generally recorded under the intercropping system. In contrast, greater weed species diversity and richness were observed in the intercropping treatment at all assessment dates (Table S5).

### Beekeeping value of buckwheat and pollinator abundance under various cultivation systems

**Fig. 4:**
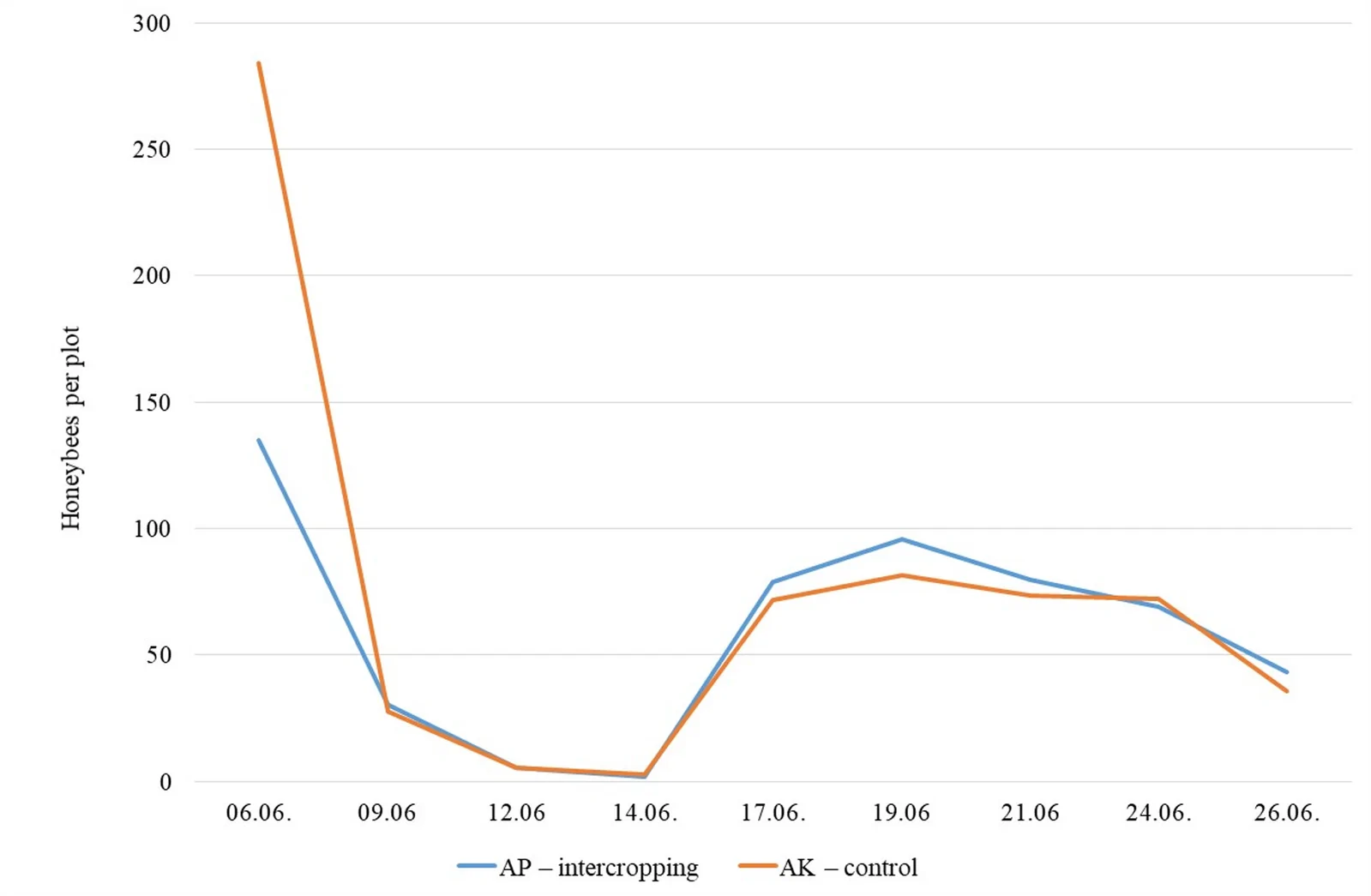
Temporal changes in honeybee abundance in the intercropping (AP) and control (AK) systems during the buckwheat flowering period.

Based on the conducted research, the mass of sugars from 10 buckwheat flowers, the average number of flowers per plant the number of flowers per hectare, and sugar availability tended to be higher under the intercropping system in 2024 (Table 4). Although the differences were not statistically significant, these results suggest that intercropping did not reduce the attractiveness of buckwheat to pollinators. The average number of honeybees recorded during the entire observation period was slightly higher in the control treatment (AK). However, temporal fluctuations in pollinator abundance were observed during the flowering period, and on several observation dates higher numbers of honeybees were recorded in the intercropping system (Fig. 4).

**Table 4:** Beekeeping value of buckwheat under intercropping conditions in 2024 (mean ± SD).

Specification	2024	
	AP	AK
Mass of sugars of 10 flowers of buckwheat (mg)	0.43 ± 0.13	0.41 ± 0.17
Mass of nectar of 10 flowers of buckwheat (mg)	3.03 ± 1.43	3.11 ± 2.17
Average number of flowers on 1 plant per 1 day of observation	15.65 ± 2.97	13.51 ± 3.02
The average number of flowers million/1 ha^−1^ per day of observation	39.13 ± 7.43	33.77 ± 7.56
Availability of raw sugar in kg per 1 ha	1.74 ± 0.67	1.44 ± 0.75
Average number of honeybees for 1 block (pcs.)	55.93 ± 45.49	72.76 ± 84.87

*AK* control (buckwheat monoculture), *AP* intercropping (*Paulownia*–buckwheat intercropping system). *SD* standard deviation.

In the final week of measurements, solitary bees were also recorded, although their abundance remained low (1.33 and 1.29 individuals per block for AP and AK, respectively). Single butterflies, mainly *Pieris* sp., were occasionally observed but were not included in the analysis because of their low abundance.

### Presence of bacteria and fungi in the soil under the intercropping system

#### Temporal changes in fungal root colonization under the *Paulownia* system

Intercropping significantly increased total bacterial abundance, dehydrogenase activity (DHA), and T-GRSP concentration compared with the control treatment, whereas no significant effect was observed for fungal abundance (Table 5). Microbial abundance also varied depending on the sampling date, with the highest bacterial and fungal counts recorded at the final sampling term (T2). In contrast, DHA activity remained relatively stable throughout the growing season, while the highest T-GRSP concentration was observed during buckwheat flowering (T1b).

**Table 5:** Total abundance of bacteria and fungi, dehydrogenase activity (DHA), and total glomalin-related soil protein (T-GRSP) concentration under different cultivation systems and sampling dates.

Samples	Total bacterial count (CFU g^−1^)	Total fungal count (CFU g^−1^)	DHA (µg TPF g^−1^ h^−1^)	T-GRSP (µg g^−1^)
AP	5.74 ± 0.60 × 10^6^ a	4.92 ± 0.91 × 10^4^ a	5.34 ± 0.25 a	2289.34 ± 91.28 a
AK	4.28 ± 1.05 × 10^6^ b	4.80 ± 0.44 × 10^4^ a	4.61 ± 0.06 b	2172.82 ± 112.14 b
T1	3.58 ± 1.21 × 10^6^ b	4.54 ± 0.62 × 10^4^ b	4.86 ± 0.19 a	2231.11 ± 120.58 b
T1b	5.06 ± 0.47 × 10^6^ ab	4.02 ± 0.09 × 10^4^ ab	4.89 ± 0.24 a	2398.68 ± 8.07 a
T2	6.40 ± 0.47 × 10^6^ a	6.03 ± 0.71 × 10^4^ a	5.16 ± 0.68 a	2063.5 ± 46.05 c

*AK* control, *AP* intercropping, T1, T1b, T2—the first, second and third soil sampling dates; DHA—dehydrogenase activity expressed as the amount of released μg triphenylformazan (TPF) per hour per gram of soil; GRSP—glomalin-related soil protein content per gram of air-dried soil. Values followed by different letters in columns indicate significant differences according to two-way ANOVA with post-hoc Tukey HSD test, ± SE (standard error).

### Identification of bacterial and fungal phylum in cultivation system

**Fig. 5:**
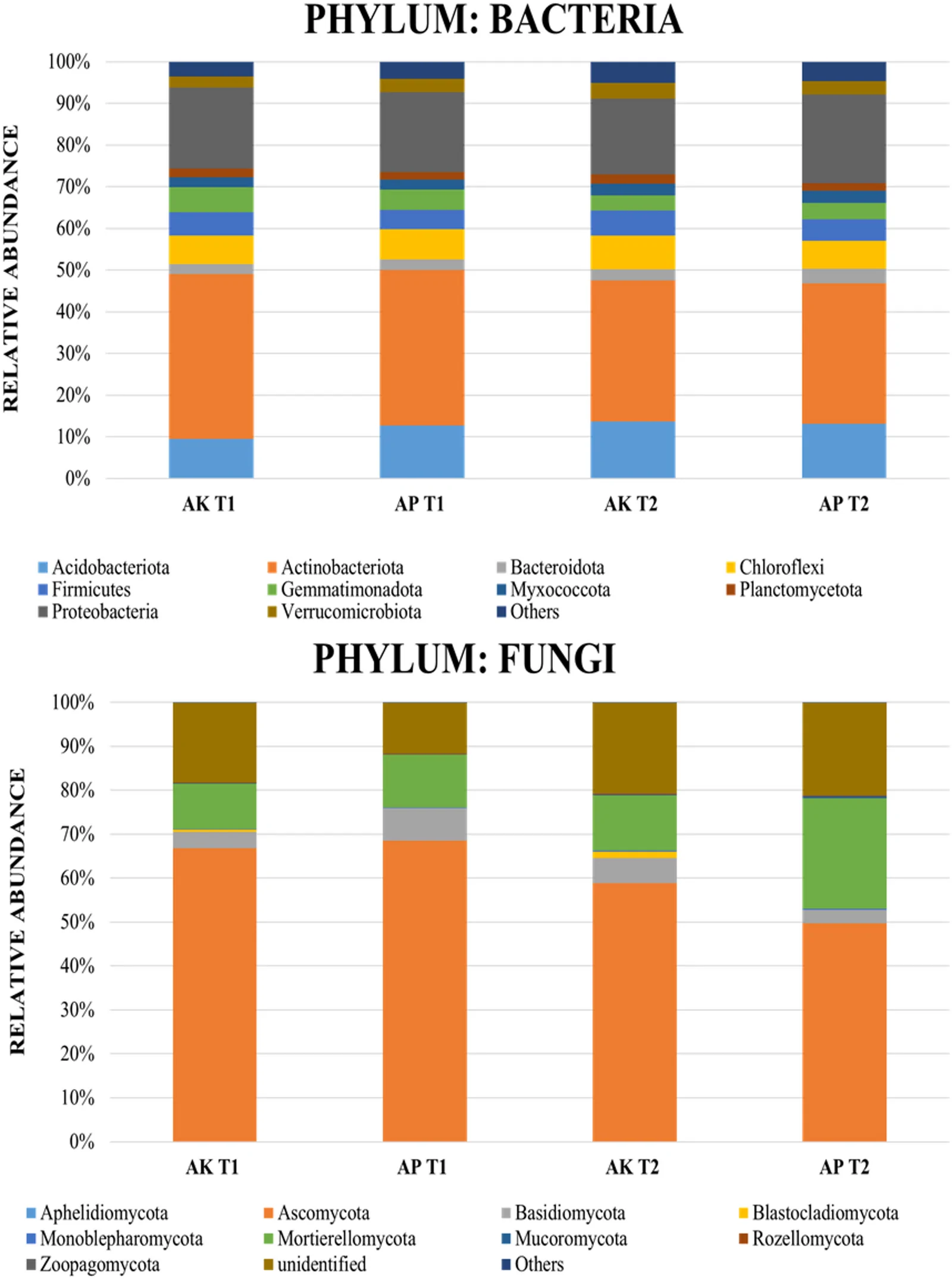
Relative abundance of bacterial and fungal phyla in the control (AK) and intercropping (AP) systems at two soil sampling dates (T1 and T2).

Soil samples collected from both intercropping (AP) and control (AK) systems were dominated by bacterial phyla including Actinobacteriota, Proteobacteria, Acidobacteriota, Chloroflexota, and Firmicutes (Fig. 5). Actinobacteriota and Proteobacteria represented the most abundant bacterial groups in all treatments and sampling dates. Temporal changes in the relative abundance of selected bacterial phyla were observed between sampling dates; however, differences between cultivation systems were generally moderate.

Raw microbiome sequencing data are available in the NCBI Sequence Read Archive under BioProject accession number PRJNA1260210.

Soil fungal communities in both cultivation systems were dominated by Ascomycota, Mortierellomycota, and Basidiomycota (Fig. 5). Ascomycota represented the most abundant fungal phylum across all treatments, although its relative abundance decreased at the second sampling date. In contrast, Mortierellomycota showed increased abundance during buckwheat flowering, particularly in the intercropping system.

Raw mycobiome sequencing data are available in the NCBI Sequence Read Archive under BioProject accession number PRJNA1260755.

### Major fungal groups colonizing buckwheat roots

**Fig. 6:**
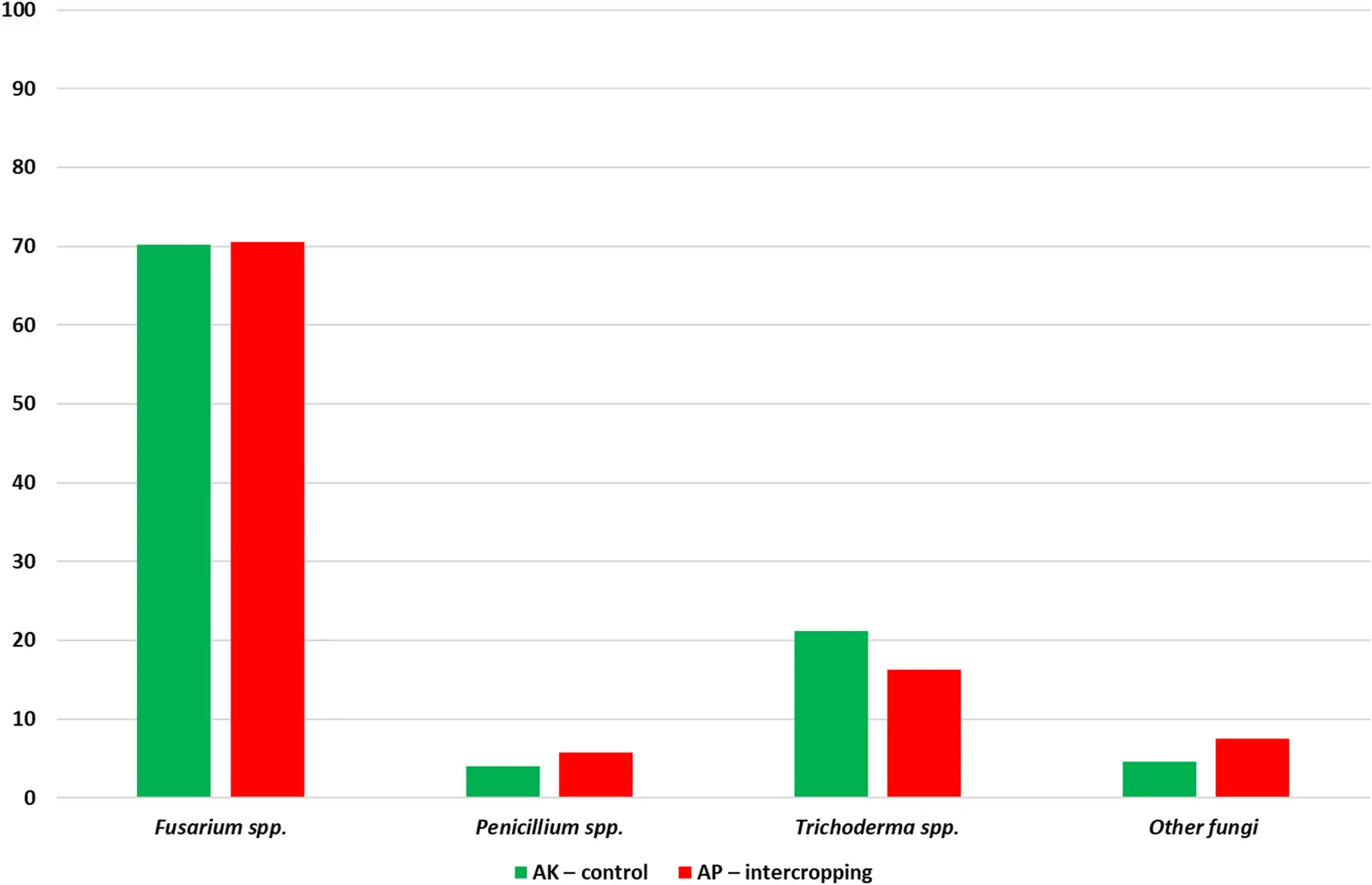
Percentage of filamentous fungi isolated from buckwheat roots using the culture method.

Fungal communities colonizing buckwheat roots were dominated by *Fusarium* spp., particularly *F. oxysporum*, in both cultivation systems (Fig. 6, Table S6). The abundance of *Trichoderma* spp. was slightly higher in the control treatment (AK) than in the intercropping system (AP). *Penicillium* spp. and other fungal groups, including *Phoma* spp. and *Exserohilum* spp., occurred at relatively low abundance (Table S6).

### Effect of intercropping on biometric traits and the yield

Most morphological and flowering-related traits of buckwheat did not differ significantly between cultivation systems (Table 6). However, plants grown under intercropping conditions generally showed higher values for branching, number of inflorescences, and number of full seeds. A higher number of full seeds was observed in the intercropping treatment, although the difference was not statistically significant.

**Table 6:** Selected morphological and flowering biology-related traits of buckwheat (averages for specific treatments) in 2024.

Specification	Plant height (cm)	Number of branches on main shoot	Number of branches	Number of inflorescences	Number of full seeds
AP	44.7	0.80	1.82	4.56	24.0
AK	46.2	0.46	1.44	4.00	19.3
Student’s t-test	ns—non-significant * p* = 0.60	ns—non-significant * p* = 0.08	ns—non-significant * p* = 0.07	ns—non-significant * p* = 0.32	ns—non-significant * p* = 0.22

*AK* control (buckwheat monoculture), *AP* intercropping (*Paulownia*–buckwheat intercropping system).

No significant differences were observed for seed yield or its structural components between cultivation systems (Table 7), although slightly higher values for the number of full seeds and total seed mass per plant were recorded under intercropping conditions.

**Table 7:** Seed yield and yield components (average for combination) in the 2024 season.

Specification	Number of plants before harvest per m^-2^	Number of full seeds	Total seed mass per plant (g)	Buckwheat yield (t ha^-1^)
AP	231.8	18.1	0.40	0.43
AK	242.6	14.7	0.30	0.44
Student’s *t* test	ns—non-significant *p* = 0.39	ns—non-significant *p* = 0.21	ns—non-significant *p* = 0.17	ns—non-significant *p* = 0.63

*AK* control (buckwheat monoculture), *AP* intercropping (*Paulownia*–buckwheat intercropping system).

At the end of the 2024 growing season, the average height of *Paulownia* trees was 514 cm. Mean crown volume measured in October was 0.12 m^3^, while trunk circumference at breast height reached 26.4 cm. *Paulownia* flowered for the first time in the fifth year after planting; however, flowers were damaged by frost events in April 2024.

## Discussion

The experiment primarily focused on the impact of intercropping on weed communities (segetal flora) and soil mesofauna, with particular emphasis on differences in species composition, abundance, and biodiversity under the *Paulownia*–buckwheat agroforestry system. Additional analyses of soil microorganisms and pollinators were included as complementary components providing broader ecological context for agroecosystem functioning. The results suggest that intercropping may promote greater biodiversity through increased habitat heterogeneity and interactions among agroecosystem components, although not all observed differences were statistically significant.

### Biodiversity of soil mesofauna

As a result of the common cultivation of plants from different botanical groups, the abundance of soil organisms may increase. It is understandable that the presence of plants is one of the important factors influencing their occurrence ^17,18^. In assessing the effect of intercropping, springtails and mites are often used, as they are the most numerous representatives of soil mesofauna ^19,20^. The number of Acari in our study was higher in the intercropping system compared to monoculture. However, no significant increase in the number of Collembola was observed in this cultivation system.

Soil mesofauna respond relatively rapidly to environmental changes and are widely recognized as bioindicators of soil biological quality ^21,22^. In particular springtails and mites contribute to organic matter decomposition, nutrient cycling, and improvement of soil structure and porosity. Collembola may additionally influence water infiltration and root penetration within the soil profile ^23^. Such effects may be expected in buckwheat cultivation systems established in the vicinity of *Paulownia* trees. In our investigation, Collembola diversity was higher in the intercropping system, although differences in abundance were not significant. Greater plant diversity in unmanaged sites adjacent to agricultural fields, including woody plants, generally has a positive effect on the species composition of Collembola ^24^. Some springtail species may also reduce the occurrence of pathogenic fungi by regulating soil microflora ^25^. Overall, intercropping system created more favourable conditions for maintaining soil mesofauna biodiversity and supporting ecological stability within the agroecosystem ^26–28^.

### Biodiversity of weeds

High biodiversity of segetal flora is currently considered an important component of sustainable agricultural landscapes, particularly in regions affected by intensive agricultural practices ^29,30^. In the present study, the dominant weed species included *Chenopodium album*, *Echinochloa crus-galli*, and *Agropyron repens*, which is consistent with previous reports from buckwheat cultivation in Poland ^31^. Although no statistically significant differences in weed species richness were observed between cultivation systems, weed biomass tended to be higher in the monoculture treatment (AK) than in the intercropping system (AP).

Weed competition is particularly important during the early growth stages of buckwheat and may influence soil water availability ^32,33^. In our study, lower soil moisture and higher weed biomass were observed in the monoculture system, suggesting greater competition for water resources. At the same time, many weed species recorded in the experiment, including *Centaurea cyanus*, *Taraxacum officinale*, and *Papaver rhoeas*, may provide ecological benefits by supporting pollinators and increasing agroecosystem biodiversity ^9,29^. These findings suggest that weed communities may simultaneously function as both competitive and ecologically beneficial components of intercropping systems.

### Beekeeping value of buckwheat and pollinators abundance

Previous studies have shown that pollinator biodiversity and activity may increase in diversified agricultural systems compared with conventional monocultures ^12,34^. Buckwheat is considered an important melliferous plant due to its entomophilous pollination and high nectar production ^35–37^. In our experiment, the highest nectar and sugar production, as well as the greatest abundance of honeybees, were observed during the initial flowering period, whereas less favorable weather conditions later reduced pollinator activity and nectar availability.

Honeybees were the dominant pollinators observed throughout the study, while solitary bees, butterflies, and bumblebees occurred only occasionally. The presence of flowering weeds and intercropping vegetation may additionally support pollinator diversity by increasing nectar and pollen availability ^12,38^. The greater diversity of flowering plants observed in the intercropping system may therefore contribute to maintaining pollinator activity and increasing habitat heterogeneity within the agroecosystem. These findings suggest that the *Paulownia*–buckwheat intercropping system may support pollinator activity and broader agroecosystem biodiversity.

### Biodiversity of soil microorganisms

Intercropping systems may enhance soil microbial abundance and diversity through changes in soil properties, root exudates, and organic matter accumulation, thereby supporting beneficial microorganisms involved in nutrient cycling and carbon transformation ^1,39^. Previous studies demonstrated that intercropping may increase microbial diversity and alter microbial community structure compared with monoculture systems ^40–43^. Tree-based intercropping systems may additionally create more heterogeneous root and vegetation patterns, contributing to greater microbial diversity ^44^.

In our study, Actinobacteriota and Proteobacteriota were the dominant bacterial phyla identified in both cultivation systems, which is consistent with previous reports from agricultural soils ^41,45^. Intercropping also affected fungal community structure, including an increase in the relative abundance of Chytridiomycota and a decrease in Basidiomycota. The dominance of Actinobacteriota observed in the present study may additionally be associated with the slightly acidic soil pH (5.8–6.2).

Soil moisture is an important factor shaping microbial diversity and soil biochemical activity ^46–48^. In our study, higher soil moisture in the intercropping system may have contributed to increased biological activity, microbial abundance, and higher T-GRSP concentration. Increased glomalin content may additionally indicate improved soil aggregation and stabilization processes under intercropping conditions. However, soil sampling date did not significantly affect DHA activity.

### Effect of intercropping on the buckwheat yield and its components

Plant height, branching, and yield are important indicators of buckwheat growth and competitiveness ^49^. In our study, biometric traits and yield were not significantly affected by the cultivation system, although slightly higher values were observed under intercropping with *Paulownia*. This may result from complementary interactions between species and favourable shading conditions that can stimulate crop growth ^50,51^. Similar observations were reported by Thevathasan and Gordon (2004) ^52^, who found that yields of C3 crops in tree-based intercropping systems were comparable to or higher than those in monocultures. Many studies have confirmed the positive effects of intercropping on crop productivity and profitability ^10,53,54^, although some crop combinations may reduce yields because of interspecific competition ^55,56^.

In our experiment, no negative effect of intercropping on buckwheat yield or yield components was observed, which was also consistent with results obtained in 2021–2023 ^11^. In 2024, buckwheat yield was relatively low (0.44 t·ha^−1^), most likely due to low rainfall during April–July. Soil moisture was generally higher in intercropped plots, probably because shading by *Paulownia* leaves reduced evaporation and water stress ^57^. These findings indicate that buckwheat–*Paulownia* intercropping enables efficient use of the space between tree rows without negatively affecting nectar production or crop yield.

### Study limitations and future perspectives

Although the obtained results indicate a beneficial effect of buckwheat–*Paulownia* intercropping on agroecosystem biodiversity, some of the observed differences were not statistically significant. This applied, among others, to buckwheat yield, Collembola abundance, weed abundance, and pollinator activity, suggesting that the effects of intercropping may strongly depend on weather conditions and the duration of the agroforestry system. The relatively low buckwheat yield recorded in 2024 was most likely associated with unfavourable weather conditions, particularly low precipitation during the April–July period, which may have limited the productivity potential of the studied system.

A slight reduction in the abundance of *Trichoderma* spp. was also observed under intercropping conditions, indicating that changes in the soil microbiome may not always be uniformly beneficial. In addition, although intercropping increased weed diversity, it did not significantly reduce weed abundance, suggesting that further optimization of weed management practices may be necessary for the practical implementation of such systems.

It should also be emphasized that the study was conducted under the specific environmental conditions of south-western Poland and within a relatively young agroforestry system. Therefore, further long-term studies conducted under different climatic and soil conditions are required to confirm the stability and broader applicability of the observed effects.

## Conclusions

The results indicate that *Paulownia*–buckwheat intercropping may enhance selected components of agroecosystem biodiversity while maintaining stable crop productivity. The intercropping system increased the diversity of soil mesofauna and segetal flora, while many accompanying weed species additionally supported pollinator activity and habitat heterogeneity.

Intercropping also positively affected selected microbiological parameters and soil moisture conditions without negatively influencing buckwheat yield or biometric traits. Pollinator activity and nectar productivity tended to be higher under intercropping conditions.

Overall, the *Paulownia*–buckwheat intercropping system may represent a promising strategy for supporting biodiversity and sustainable agricultural production under the environmental conditions of southwestern Poland.

## Supplementary Information

Below is the link to the electronic supplementary material.

Supplementary Material 1

## Data Availability

All data generated or analysed during this study are included in this published article. Experimental research and field studies on plants (either cultivated or wild), including the collection of plant material are complying with relevant institutional, national, and international guidelines and legislation. We did not use endangered plant species for the experiments.
