# Supplementary material for: The impact of Paulownia–buckwheat intercropping on the biodiversity of different living biota
Source: Sci Rep. 2026 Jun 20;16:28315. doi: 10.1038/s41598-026-58572-1 (PMC13558687; doi:10.1038/s41598-026-58572-1)
Supplement: Supplementary file 1 [file 41598_2026_58572_MOESM1_ESM.docx]

**Buckwheat and *Paulownia* development phases**

High temperatures in the initial period of development (April–June) accelerated the development of buckwheat (Table S1). However, high temperatures and drought, especially during the flowering phase, are harmful because they can cause flower abortion. During the flowering period, it struggled with heat and low humidity, which resulted in a deterioration of nectar production. This resulted in worse bee flights. The shortening of the early development stages of buckwheat (including flowering) and poor growth caused by drought could be the reason for the low buckwheat yield. Excessive rainfall in August delayed the ripening and harvesting of buckwheat. For *Paulownia*, although temperatures were favourable for plants of the C4 cycle (*Paulownia*), there were frosts 23/24. April disrupted the development of this tree and delayed the resumption of vegetation. However, the long autumn extended the vegetation period again. All in all, the growing season for *Paulownia* turned out to be long considering Polish conditions.

**Table S1**. Oxytree and buckwheat phenological phases in 2024.

| Date |  | Number of days since resumption of growth |  | Number of days since sowing |
| --- | --- | --- | --- | --- |
|  | *Paulownia* phase |  | Buckwheat phase  BBCH |  |
| 01/04/2024 | resumption of vegetation |  | – | – |
| 08/04/2024 | development of leaf buds | 7 | – | – |
| 11/04/2024 | flowering | 10 | – | – |
| 19/04/2024 | flowering | 18 | Sowing BBCH 00 | – |
| 24/04/2024 | frozen flowers and leaves | 23 | closed cotyledons BBCH 09/10 | 5 |
| 09/05/2024 | re-development of leaf buds | 39 | open cotyledons BBCH 10 | 15 |
| 15/05/2024 | development of leaf buds | 45 | 2–3 true leaves BBCH 12 | 21 |
| 20/05/2024 | development of leaf buds | 50 | flower buds BBCH 50 | 26 |
| 04/06/2024 | crown growth | 70 | full bloom BBCH 63 | 31 |
| 11/06/2024 | crown growth | 76 | full bloom BBCH 65 | 38 |
| 17/06/2024 | crown growth | 82 | brown seeds BBCH 80 | 51 |
| 23/07/2024 | crown growth | 118 | brown seeds BBCH 85 | 88 |
| 23/08/2024 | setting flower buds | 149 | Harvest BBCH 89 | 120 |
| 06/11/2024 | leaves fall (end of vegetation phase) | 235 | – | – |

**Table S2.** Number (no./m^2^) of dominant weed species after buckwheat **emergence (BBCH 12)** in 2024.

| Specification | *Chenopodium album* | *Echinochloa crus-galli* | *Agropyron repens* |
| --- | --- | --- | --- |
| AP | 21.2 | 14.4 | 10.5 |
| AK | 26.8 | 22.8 | 4.6 |
| Student’s t-test | ns – statistically insignificant  p=0.37 | ns – statistically insignificant  p=0.14 | ns –  non-significant  p=0.08 |

AK – control (buckwheat monoculture); AP – intercropping (Paulownia–buckwheat intercropping system).

**Table S3.** Number (no./m^2^) and dry mass (g) of dominant weed species **at buckwheat stage BBCH 85** in 2024.

| Specification | *Chenopodium album* | | *Echinochloa crus-galli* | | *Setaria viridis* | |
| --- | --- | --- | --- | --- | --- | --- |
|  | no. | g | no. | g | no. | g |
| AP | 39.2 | 63.5 | 15.4 | 10.4 | 13.0 | 13.0 |
| AK | 46.2 | 144.1 | 17.6 | 19.1 | 6.0 | 5.9 |
| Student’s t-test | ns – non statistically significant  p=0.40 | ns – non statistically significant  p=0.16 | ns – non statistically significant p=0.70 | ns – non statistically significant  p=0.16 | ns – non statistically significant  p=0.17 | ns – non statistically significant  p=0.46 |

AK – control (buckwheat monoculture); AP – intercropping (Paulownia–buckwheat intercropping system).

**Table S4.** Number (no./m^2^) and dry mass (g) of dominant weed species **after buckwheat harvest** in 2024.

| Specification | *Chenopodium album* | | *Echinochloa crus-galli* | | *Setaria viridis* | |
| --- | --- | --- | --- | --- | --- | --- |
|  | no. | g | no. | g | no. | g |
| AP | 7.2 | 1.4 | 16.6 | 5.8 | 6.0 | 3.4 |
| AK | 5.5 | 0.9 | 8.5 | 15.9 | 6.8 | 7.6 |
| Student’s t-test | ns – non statistically significant p=0.67 | ns – non statistically significant p=0.69 | ns – non statistically significant  p=0.37 | ns – non statistically significant  p=0.14 | ns – non statistically significant  p=0.77 | ns – non statistically significant  p=0.17 |

AK – control (buckwheat monoculture); AP – intercropping (Paulownia–buckwheat intercropping system).

**Table S5.** List of all weed species determined at the three dates of buckwheat cultivation.

| Weed species | Buckwheat emergence (BBCH 12) | | Buckwheat at (BBCH 85) | | After buckwheat harvest | |
| --- | --- | --- | --- | --- | --- | --- |
|  | AP | AK | AP | AK | AP | AK |
| *Chenopodium album* | +++ | +++ | +++ | +++ | + | ++ |
| *Echinochloa crus-galli* | +++ | +++ | +++ | +++ | ++ | - |
| *Agropyron repens* | +++ | ++ | - | ++ | ++ | ++ |
| *Setaria viridis* | - | - | +++ | ++ | +++ | ++ |
| *Erodium cicutarium* | + | + | ++ | - | ++ | ++ |
| *Amaranthus retroflexus* | ++ | ++ | ++ | ++ | ++ | ++ |
| *Thlaspi arvense* | + | + | - | - | ++ | + |
| *Anchusa arvensis* | + | - | + | - | - | - |
| *Geranium pusillum* | + | + | - | - | + | + |
| *Poa annua* | + | - | - | - | - | - |
| *Equisetum arvense* | + | - | + | - | - | + |
| *Galium aparine* | + | - | - | - | - | - |
| *Bromus sp.* | + | - | - | - | - | - |
| *Senecio vulgaris* | + | - | - | - | - | - |
| *Centaurea cyanus* | + | - | - | - | - | - |
| *Myosotis arvensis* | - | - | - | - | ++ | - |
| *Viola arvensis* | - | - | - | - | ++ | + |
| *Taraxacum officinale* | - | - | - | - | + | + |
| *Sonchus arvensis* | - | - | - | - | + | - |
| *Erigeron canadensis* | - | - | - | - | + | - |
| *Veronica hederifolia* | - | - | - | - | + | - |
| *Stellaria media* | - | - | - | - | ++ | - |
| *Galinsoga parviflora* | - | - | - | - | ++ | ++ |
| *Capsella bursa-pastoris* | - | - | - | - | ++ | + |
| *Cirsium arvense* | - | - | - | - | + | - |
| *Digitaria sanguinalis* | - | - | - | - | ++ | ++ |
| *Papaver rhoeas* | - | - | - | - | ++ | + |
| *Polygonum aviculare* | - | - | - | - | + | + |
| *Artemisia vulgaris* | - | - | - | - | + | ++ |
| *Spergula arvensis* | - | - | - | - | - | ++ |

AK – control (buckwheat monoculture); AP – intercropping (Paulownia–buckwheat intercropping system). For all weed species, three ranges were adopted relating to their abundance on treatments with and without *Paulownia*:0.1–1.0 (+); 1.1–10.0 (++); 10.1–46.2 (+++)

**Table S6**. Percentage of filamentous fungi isolated from buckwheat roots using the culture method.

| Total number of colonies (sum) | AK | AP |
| --- | --- | --- |
|  | 373 | 333 |
| *Colletotrichum* sp. | 0.9 (3±0.02) | 0.6 (2±0.12) |
| *Exserohilum pedicellatum* | 0.6 (2±0.12) | 0.9 (3±0.12) |
| *Fusarium culmorum* | 1.5 (5±0.16) | 0.9 (3±0.20) |
| *Fusarium oxysporum* | 76.3 (254±0.94) | 69.1 (230±0.99) |
| *Fusarium sporotrichoides* | 0.9 0.9 (3±0.12) | 0.6 (2±0.12) |
| *Mucor mucedo* | 1.8 (6±0.19) | 2.4 (8±0.25) |
| *Penicillium notatum* | 0 | 0.6 (2±0.12) |
| *Penicillium purpurogeum* | 1.8 (6±0.19) | 1.2 (4±0.19) |
| *Penicillium* sp. | 2.7 (9±0.37) | 3.9 (13±0.51) |
| *Phoma* sp. | 0.9 (3±0.20) | 0.6 (2±0.12) |
| *Rhizopus nigricans* | 0.6 (2±0.12) | 1.5 (5±0.27) |
| *Trichoderma hamatum* | 1.5 (5±0.16) | 1.5 (5±0.16) |
| *Trichoderma harzianum* | 21.3 (71±0.24) | 14.7 (49±0.60) |
| Other fungi | 1.2 (4±0.19) | 1.5 (5±0.32) |

AK – control (buckwheat monoculture); AP – intercropping (Paulownia–buckwheat intercropping system).

( ) – total number of colonies of fungi and ± SE, standard error
